# reGenotyper: Detecting mislabeled samples in genetic data

**DOI:** 10.1371/journal.pone.0171324

**Published:** 2017-02-13

**Authors:** Konrad Zych, Basten L. Snoek, Mark Elvin, Miriam Rodriguez, K. Joeri Van der Velde, Danny Arends, Harm-Jan Westra, Morris A. Swertz, Gino Poulin, Jan E. Kammenga, Rainer Breitling, Ritsert C. Jansen, Yang Li

**Affiliations:** 1 Groningen Bioinformatics Centre, University of Groningen, Groningen, The Netherlands; 2 Laboratory of Nematology, Wageningen University, Wageningen, The Netherlands; 3 Faculty of Life Sciences, University of Manchester, Manchester, United Kingdom; 4 Genomics Coordination Center, University of Groningen, University Medical Center Groningen, Groningen, The Netherlands; 5 Divisions of Genetics and Rheumatology, Department of Medicine, Brigham and Women’s Hospital and Harvard Medical School, Boston, Massachusetts, United States of America; 6 Partners Center for Personalized Genetic Medicine, Boston, Massachusetts, United States of America; 7 Program in Medical and Population Genetics, Broad Institute of MIT and Harvard, Cambridge, Massachusetts, United States of America; 8 Manchester Institute of Biotechnology, Faculty of Life Sciences, University of Manchester, Manchester, United Kingdom; 9 University of Groningen, University Medical Center Groningen, Department of Genetics, Groningen, The Netherlands; Fred Hutchinson Cancer Research Center, UNITED STATES

## Abstract

In high-throughput molecular profiling studies, genotype labels can be wrongly assigned at various experimental steps; the resulting mislabeled samples seriously reduce the power to detect the genetic basis of phenotypic variation. We have developed an approach to detect potential mislabeling, recover the “ideal” genotype and identify “best-matched” labels for mislabeled samples. On average, we identified 4% of samples as mislabeled in eight published datasets, highlighting the necessity of applying a “data cleaning” step before standard data analysis.

## Introduction

With the development of a wide range of high-throughput molecular profiling methods in recent years, there has been significant growth in the number of genetic studies on molecular profiles from genetically different individuals [1–6], aiming at the identification of the functional consequences of naturally-occurring and induced genetic variation (these studies are often referred to as genetical genomics [7,8] or expression genetics [9] experiments). Statistical significance testing is used to suggest causal relationships between genetic variation (polymorphisms) at the genomic level and phenotypic variation at multiple molecular levels (transcriptomics, proteomics, and/or metabolomics) using methods such as quantitative trait locus (QTL) mapping and genome-wide association studies (GWAS). Obviously, the effectiveness of significance testing depends critically on the accurate labeling of the samples, i.e., the genotype data used for statistical testing needs to correctly represent the true genotypes of the examined individuals. However, samples can be wrongly labeled in the laboratory due to human error, resulting in the assignment of wrong genotypes to individual samples; as we show below, this is a surprisingly common phenomenon [10].

A wrong genotype assignment will seriously weaken the significance testing [11] in genetical genomics studies on model organisms, especially for experiments with relatively small sample sizes. Technical genotyping errors (e.g. assigning incorrect SNP nucleotide) may also impact statistical power of genomics studies. However, these can be treated by some of the QTL mapping tools [12,13]. Particularly, mislabeled samples will influence the detection of small genetic effects. This could, on the one hand, explain the lack of consistent results across some experiments reported in meta-analyses of genetical genomics studies [14]. On the other hand, mislabeled samples could also lead to false positives for QTL mapping or GWAS in certain conditions. For example, when performing multiple QTL mapping [15], the mapping of the second (minor) QTL is based on the residuals from the phenotypes after correction for the first (major) QTL effect [16]. When there are mislabeled samples in the data, this correction process is actually turning these samples into outliers, which subsequently results in a (slight) increase in number of significant minor QTLs. Obviously, these extra minor QTLs are all false positives without biological meaning. Unfortunately, permutation methods will not help to alleviate this problem since the correction for the major QTL is done after the permutation. Following a similar reasoning, the mislabeled samples can lead to false results when testing for interactions between QTLs to correct for the deviation between data and model, such as epistasis or QTL-by-environment interactions. Therefore, new methods are necessary to detect and correct mislabeled samples before the data analysis process begins and increase the power to map meaningful QTLs.

**Fig 1:**
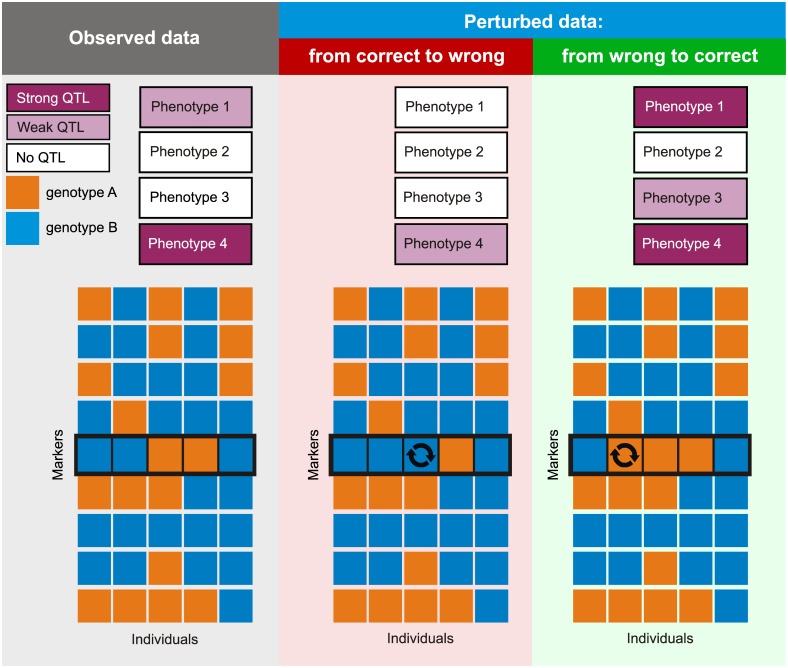
Graphical summary of the reGenotyper algorithm. reGenotyper uses a data perturbation strategy and exploits the highly parallel nature of molecular profilinrg in modern genetic studies. 1) Observed genotype data and QTLs. The data matrix in the middle contains the genotype information at each marker position (row) for each sample (column), where orange and blue represent two different genotypes. The observed QTL significance of Phenotypes 1–4 from a standard QTL mapping technique is shown in graded shades of purple, with a darker color representing a stronger QTL significance. 2) Perturbation of true genotypes. Specifically, perturbing the genotype at a particular marker of a correct sample (correct → wrong) will lead to a decreased QTL significance for all molecular traits mapping to a QTL near that marker. In this panel, the genotype of the 3^rd^ sample at the 5^th^ marker position (indicated by an arrow circle) is randomly perturbed (changed from the orange to the blue allele). Then we re-map the QTLs using the perturbed genotype data and unchanged phenotype matrix and observe that for most of the QTL the significance decreases (dark color changes to light color), i.e. the QTL loses significance if noise is added. 3) Correction of wrong genotypes. The genotype of the 2^rd^ sample at the 5^th^ marker position (indicated by an arrow circle) is randomly perturbed (changed from the blue to orange allele). Then we re-map the QTLs using the perturbed genotype data and unchanged phenotype matrix. In this case, for most of the QTL the significance increases after perturbation (light color changes to dark color) since the original genotype was wrong. When such an increase is observed for a number of phenotype—marker pairs, it suggests that the genotype of this sample was mislabeled.

Here we present *reGenotyper*, which implements a fast and accurate algorithm to identify samples that are likely to have been mislabeled, based on the measured phenotypes (Fig 1). Our approach is based on a data perturbation strategy and exploits the highly parallel nature of molecular profiling in modern genetic studies. reGenotyper aims to detect mislabeled samples in genetical genomics studies based on the heuristic “significance change value”. Our tool is able to quickly and precisely detect potential sample mislabeling using combined power of hundreds or thousands of QTLs. This makes it perfectly suited for studies based on high-throughput molecular profiling but unfeasible for traditional QTL studies based on limited number of phenotypes.

We show that the highly parallel nature of genetical genomics studies allows the sensitive and specific detection of the problematic samples. We also show that the frequency of wrongly labeled samples in such studies can be high (on average, 4% of samples are mixed up in the worm studies we analyzed [17,18], and similarly high values have been reported for human [19] and mice [10] studies [19]. As wrong sample labels seriously affect the power of QTL detection in studies that are already operating at the limit of statistical viability [20–22], the application of a mislabeled sample detection (and correction) step would be strongly recommended for any GWAS or QTL study on large-scale molecular profiling data. We implemented *reGenotyper* in the R programming language [23]. The R package and documentation are freely available from the CRAN repository and at http://www.molgenis.org/regenotyper.

## Materials and methods

For sake of simplicity, we will showcase our algorithm on transcriptome data from a population of recombinant inbred lines (RILs). RILs are homozygous individuals that result from repeated self-mating or sibling mating, starting from an F_1_ of two homozygous parents, carrying alleles of type *A* and type *B*, respectively. The genome of a RIL is therefore a mosaic of the “founder” genomes, making them a perfect population to illustrate the mode of action of our algorithm. Nonetheless, our method can be applied to any type of high-throughput phenotype data (e.g. proteomics, metabolomics) from a variety of other population types.

### reGenotyper mode of action

The reGenotyper algorithm works as follows: perturbing the genotype at a particular marker of a correct sample (correct → wrong) will lead to a decreased QTL significance (e.g. Δ*t* = *t*^*new*^ −*t*^*old*^ < 0, when using the t statistic) for all molecular traits mapping to a QTL near that marker (Fig 1). This would be true for as many molecular traits—marker combinations as there are QTLs, when the genotype of this sample was originally correctly labelled. Therefore, the distribution of Δt values for the correct sample n ($\Delta t_{k,m}^{n}$ values from K traits at M markers) would roughly show a single-component distribution, and the mean of the distribution is expected to be smaller than zero: the reason is that when perturbing the genotype of a correct sample, the significance of QTLs is expected to be reduced by the introduction of wrong marker data. In contrast, perturbing the genotype at a particular marker of a mislabeled sample (wrong → correct) will lead to an improved QTL significance (e.g. Δ*t* = *t*^*new*^ −*t*^*old*^ < 0, when using t statistic) for all molecular traits mapping to a QTL near that marker (Fig 1). This would be true for as many molecular traits—marker combinations as there are QTLs, when the genotype of this sample was originally mislabeled. Therefore, when a sample has been mislabeled, the distribution of Δt values for this sample n ($\Delta t_{k,m}^{n}$ values from K traits at M markers) would show a mixture distribution, with one extra component with a mean larger than 0. Although Δt for a certain trait at a certain marker might show a non-zero value simply by chance, the identification of mislabeled samples is based on the Δt values from a large number of traits measured in parallel (e.g., the top x QTLs at one specific marker) at a number of marker positions (different and largely independent sets of QTLs at those markers), thus making use of the data-richness of genetical genomics experiments. Therefore, the distribution of all $\Delta t_{k,m}^{n}$ (*k* = 1,…,*K*;*m* = 1,…,*M*) from one sample can actually be used to evaluate the chance that a sample is mislabeled. reGenotyper also provides an estimate of the “ideal” genotype of the potential mislabeled sample and identifies the best-matched labels for each sample, from a collection of potential genotypes. The mathematical details of the algorithm and the significance assessment are provided in the S1 File.

### Test datasets

We showcase reGenotyper as a method to detect and correct mislabeled samples using five previously published [17,18] and two unpublished *C*. *elegans* datasets. The recombinant inbred lines (RILs) used in these studies were derived from *C*. *elegans* wild-types N2 and CB4856. In the temperature experiment [17], genome-wide gene expression levels of 80 RILs were profiled at two conditions (16°C and 24°C). In the aging experiment [18], gene expression profiles of 35 RILs were measured at three stages (40 h, 96 h and 214 h). In a more recent unpublished experiment on lines from the same collection, gene expression levels of 46 and 38 RILs were profiled in two different conditions. In total, 200 different RILs were used, with considerable overlap between the studies, and the lines were SNP-genotyped with an average density of 1 marker per centi-Morgan. All the published datasets can be accessed using WormQTL [www.wormqtl.org] [24] or WormQTLHD [www.wormqtl-hd.org] [25] web portals.

## Results

### reGenotyper has high power to detect mislabeled samples

The ability of reGenotyper to detect wrongly labelled samples will obviously depend on how similar the assumed (wrong) genotype is to the real genotype. We first evaluated this effect through simulation using the area under the (receiver operating characteristic) curve (AUC) approach. Specifically, we studied the performance of the method when the original true genotypes showed different proportions of common marker genotypes with the mistakenly used genotype. The more similar the mistakenly used genotype is to the correct genotype, the more difficult it should be to identify the sample as mislabeled. In S2b Fig we can see that the AUC is around 0.8 when the true genotype shares 90% of its marker genotypes with the mislabeled sample, although in real datasets such a high level of genetic likeness is extremely rare. In the case of mouse recombinant inbred lines, the probability that two randomly selected lines have the same genotypes in 18 out of 20 chromosomes (i.e. high similarity = 90%) can be roughly estimated to be 0.5^18^ = 3.8×10^−6^. With increasing dissimilarity between the two genotypes, the performance increases to an AUC of almost 100%, i.e. a perfect detection of all wrongly labeled samples.

### reGenotyper detects mislabeled samples in seven sets of expression genetics data from worm studies

**Fig 2:**
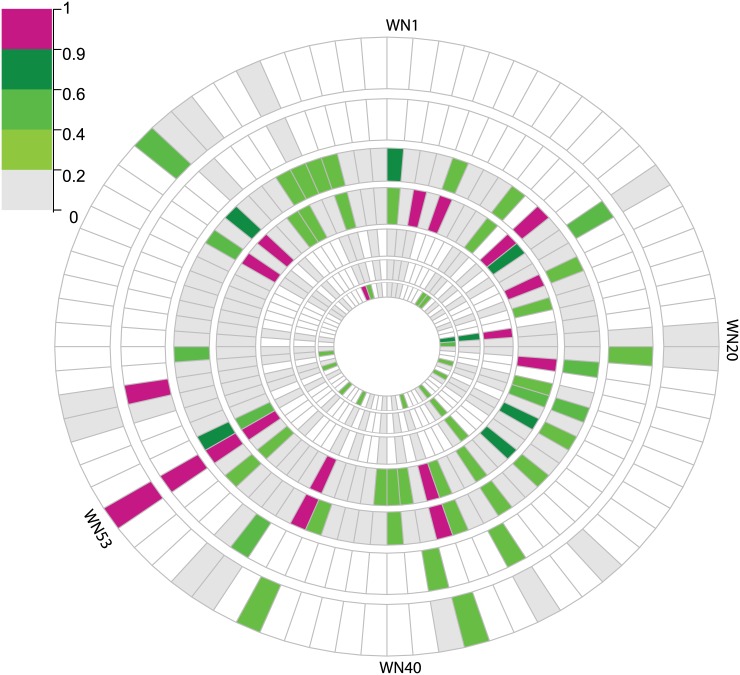
Individual evidence of the samples (arranged around the circle) being detected as potentially mislabeled sample (MS) across seven different experiments (each represented by a circle) from *C*. *elegans* studies using the reGenotyper method. Different shades of green represent the mislabeling score, with a darker color corresponding to a higher score, and magenta indicating that the sample has been detected as MS with a score larger than 90%. White indicates that the sample was not used in this experiment (as not all samples were used in all experiments). The samples with consistent strong evidence of being potentially mislabeled across experiments (i.e., showing high scores in multiple experiments) are more likely to indeed be mislabeled. Note that sample WN53 shows a mislabeling score larger than 0.9 in four independent experiments, making it very likely that it was indeed mislabeled, as confirmed by subsequent experiments.

We applied the reGenotyper on seven *C*. *elegans* datasets, as described in the Test Datasets subsection of the Materials and Methods (Fig 1). Four samples showed strong evidence (mislabeling score > 0.9) of being mislabeled in at least two different experiments (Fig 2 and S3 Fig). The WN53 line was the top candidate for being wrongly labeled: this line had the first rank amongst potentially mislabeled samples in all four experiments in which it had been used.

If samples have been mistakenly swapped with other samples in the same RIL collection, the “true” RIL genotype can be recovered by comparing the estimated genotype with all the known genotypes. Applying reGenotyper to the four mislabeled samples detected above, all of them show consistent candidate RILs in at least three independent experiments. Most interestingly, WN54 was identified as a candidate for being the true RIL for WN53 in all four experiments. In order to verify our findings, we re-genotyped the WN53 line using an Illumina SNP array. The genotyping results based on 96 equally spaced SNP markers clearly showed that indeed sample labeled as WN53 is actually WN54. A follow-up quantitative re-analysis of the temperature experimental data demonstrated the substantial gain from applying reGenotyper. Correcting the genotype for the 5 mislabeled samples leads to better consistency in the detected *trans*-eQTL, i.e. the number of *trans*-eQTL shared between two temperatures increased by 111% (from 93 to 190, FDR<0.05) (S4 Fig). This result indicates that correcting mislabeled samples could resolve, at least in part, the commonly observed lack of consistency in *trans*-QTL across studies [14,26].

### reGenotyper detects mislabeled samples in publicly available expression genetics data from mouse, worm and yeast studies

As we had identified a number of mislabeled samples in the worm datasets affecting the power to detect eQTLs, we applied our algorithm to several publicly available datasets for which the genotype and transcriptome data were available online.

1. Among 208 recombinant inbred advanced intercross lines from *C*. *elegans* [27], four samples (1.9%) showed a mislabeling score of >90%, based on 1000 permutations. The corrected mapping can be found in WormQTL [24] and WormQTLHD [25] portals.
2. Among 96 recombinant inbred lines from mouse [28], two samples (2%) showed strong evidence of being mislabeled (mislabeling score >90%), based on 1000 permutations.
3. None of the yeast segregants [29] was detected as mislabeled with the same threshold, but one sample showed suggestive evidence of being mislabeled (S5 Fig).

In each case, a number of additional samples were highlighted as suspicious and could be followed up experimentally when resources permit.

### reGenotyper can help to correct the mislabeled samples it detects in three user-defined ways

1. The simplest way is removing those unreliable samples from the dataset and performing the further analysis based on the remaining samples. This most conservative approach would remove dubious information from the original data, but it also leads to a decrease in the sample size and a concomitant reduction of statistical power. We performed a simulation study to show an effect of removing the samples. We used the median of the detected QTL significance distribution as a benchmark score. We removed samples in order of their chance of being mislabeled (i.e. according to their mislabeling score). This increased the benchmark score, until the point of removing samples with little evidence of being mislabeled (i.e. correct samples). (S6 Fig).
2. A possibly more efficient way of handling the mislabeled data would be to recover the true genotypes for the mislabeled samples. Not discarding molecular profiling information of the mislabeled samples could potentially lead to obtaining even more power for QTL detection. For example, in a simulated genetical genomics experiment (details can be found in the S1 File), the genotypes of six samples (10%) were manually swapped (i.e. exchanged between two samples that were both included in the dataset). The proportion of common marker genotypes between true genotype and mistakenly used one was on average 60% (i.e. the original genotype and the wrong new genotype still had, on average, 60% identical marker genotypes but differed for the remaining 40%). In 200 simulations, on average 23% additional simulated QTLs could be detected using the inferred true genotypes compared to using mislabeled genotypes (S7 Fig).
3. In the case that genotype information is available for a larger collection of many strains, reGenotyper can also identify the best-matched genotype for the detected mislabeled samples. This step helps to correct the swapping of sample labels in the lab (as showcased in results section).

## Discussion

### reGenotyper can be used to systematically examine the molecular profiling phenotype data to directly identify potentially mislabeled samples

To our best knowledge, there is no other tool capable of such a task. Even though there has been numerous attempts to address sample mix-ups detection [19,30–33]. A method called MixupMapper, was proposed to correct sample mix-ups in gene expression data for human genome-wide association studies [19]. However, it is based solely on the expression of genes which are influenced by genetic variation located near these genes (*cis*-eQTL). MixupMapper aims to detect pairs of samples for which the genotype information has been mistakenly swapped, but both genotypes have been measured and both samples have been included in the experiment. Thus, this method cannot find mislabeled samples that result from slightly more complex (but still very likely) experimental mix-ups, e.g. the use of wrong samples from a larger collection, or accidental duplicate measurements. Additionally, a Bayesian approach was reported to predict SNP genotypes based on RNA expression data, and then to match the predicted genotype to the observed genotype of individuals in large populations [30]. It makes use of the consistency of *cis*-eQTL across different tissues and therefore requires training data from one or more independent tissues; unfortunately this kind of replication is not available for most studies. Moreover, all of the above methods depend on eQTL, which limits their usage to gene expression studies, whereas our reGenotyper method could in principle be applied to any type of high-throughput phenotype data, including the increasingly popular and powerful genetic analysis of protein and metabolite profiles [4,5]. The tool is able to perform an analysis within minutes (*C*. *elegans* data) to hours (human data) on a personal computer. However, we recommend use of parallel computing (e.g. cloud services) so that more permutations can be performed increasing accuracy of reGenotyper.

### Limitations of reGenotyper

The package was built so that detection of sample mix-ups is performed quickly and accurately for large numbers of phenotypes. However, our heuristic “significance change value” approach is only valid if phenotype data is of massively parallel nature. Moreover, the package needs numerous phenotypes with considerable number of highly significant QTLs in order to perform meaningful permutation analysis. This limits reGenotyper usage to the high-throughput molecular studies where measuring thousands of phenotypes is feasible.

On the other side of the spectrum are studies generating truly big data. For example, genomic studies in humans utilize tens of millions of SNP markers [34]. Permuting a dataset with thousands of phenotypes mapped to tens of millions of markers might prove infeasible. It is possible to use our package on a High Performance Computing (HPC) cluster with an aid of specialized platform like xQTL workbench [35], but this requires computing expertise and access to an HPC cluster.

The accuracy of sample mix-up detection may also be affected by gene—environment interactions, as environmental variation induces major changes in phenotype that might not be linked to genetics. This can result in discovering false mix-ups. On the other hand, measuring phenotypes in multiple environments allows untangling environmental and genetic components of observed phenotypical variation [36]. Analyzing phenotypes from each of the environment separately with reGenotyper and then comparing the results increases the accuracy of mislabeling detection even further.

We aimed at as much automation of the workflow as possible. However, there is still a crucial step that requires user intervention. Samples are marked as mislabeled based on a user-defined threshold. Even though the threshold is statistically well-defined (see S1 File), the choice of a more or less stringent cut-off will depend on a user’s preferences (e.g, the perceived cost of missing a mislabeled sample) and expectations (e.g., the predicted frequency of mislabeled samples in a dataset).

## Supporting information

S1 Fig Results of a simulated genetical genomics experiment. Experiment without a mislabeled sample (a) and with mislabeled samples (b). Grey and red correspond to values of 0 and 1 for the S element value, respectively. The rows represent markers along the genome and the columns represent different samples. (a) S values of 1 (red spots) are scattered in the figure, indicating that no sample has been mislabeled. (b) Vertical red bars indicate that the corresponding samples (columns) are very likely to have been mislabeled. In this simulated experiment, six (~10%) mislabeled samples sharing about 50% common marker genotypes with the correct sample were included, and these were all clearly identified.
(PNG)

S2 Fig Detection of mislabelling between similar samples. a) Comparison of the distribution of Δt for a correctly labeled sample (green) and a wrongly labeled sample (magenta) from a simulated dataset. The wrongly labeled sample and the true genotype are 70% identical. b) Box plot of the area under the receiver operating characteristic curve (AUC) for the reGenotyper algorithm for different scenarios. The x-axis represents ten different situations, i.e. different proportions of common marker genotypes between the mislabeled sample and the true genotype of this sample; in the most challenging scenario, the mislabeled sample shares 95% of its marker genotypes with the true sample. Under each scenario, 50 simulations were carried out. In each simulation, a gene expression dataset of 100 samples with 10% mislabeled samples was simulated.
(PNG)

S3 Fig Individual evidence of the 200 RIL samples (column) being detected as potentially mislabeled. Sample across seven different experiments (rows) from our lab using the reGenotyper algoritm. Different shades of green gradients represent the mislabeling score. Darker green corresponds to higher scores, while magenta indicates that the sample was detected as MS with a score larger than 90%. White indicates that the sample was not selected for use in this experiment.
(PNG)

S4 Fig Impact of removal of MS on QTL mapping. Figure compares results of QTL mapping on original data (left) and after removal of 5 MS (right). Removal of MS detected by reGenotyper results in sharp increase in number of QTLs shared between two temperatures used in the study (from 93 to 190–111%).
(PNG)

S5 Fig Mislabeling scores in different experiments from a) worm, b) mouse and c) yeast. Respectively, 4 out of 208, 2 out of 96 and 0 out of 109 samples show a mislabeling score >0.9 (dashed line).
(PNG)

S6 Fig Changes in QTL significance after removing samples. The genotype and phenotype data were simulated for 60 RILs in the same way as described in the S1 File. Three (5%) mislabeled genotypes were added to the data set. The samples were removed in decreasing order of their mislabeling score. First, three mislabeled samples (MS) and later samples with little chance of being mislabeled (i.e. low mislabeling score), correct samples, (CS) were removed. The median of detected QTLs (-log10P) significance distribution (orange line) increases after removing MS and drops again after removing CS. (x axis, rm = removing).
(PNG)

S7 Fig Changes in QTL significance after recovering the true genotypes for the mislabeled samples in simulated data. 200 simulations were performed and QTL mapping was performed a) before swapping samples to simulate mislabeling, b) after swapping samples and c) after swapping samples and recovering true genotypes using reGenotyper. Plots shows rate of: red 1 –phenotypes with simulated QTL that was mapped; blue 2 –phenotypes without simulated QTL, where no QTL was mapped; green 3 –phenotypes without simulated QTL, where QTL was mapped. After recovery with reGenotyper almost all of the QTLs could be mapped correctly, compared to 77% in case with MS.
(PNG)

S1 File Detailed description of the reGenotyper algorithm. (DOC)
