# Supplementary figures and images for: reGenotyper: Detecting mislabeled samples in genetic data
Source: PLoS One. 2017 Feb 13;12(2):e0171324. doi: 10.1371/journal.pone.0171324 (PMC5305221; doi:10.1371/journal.pone.0171324)

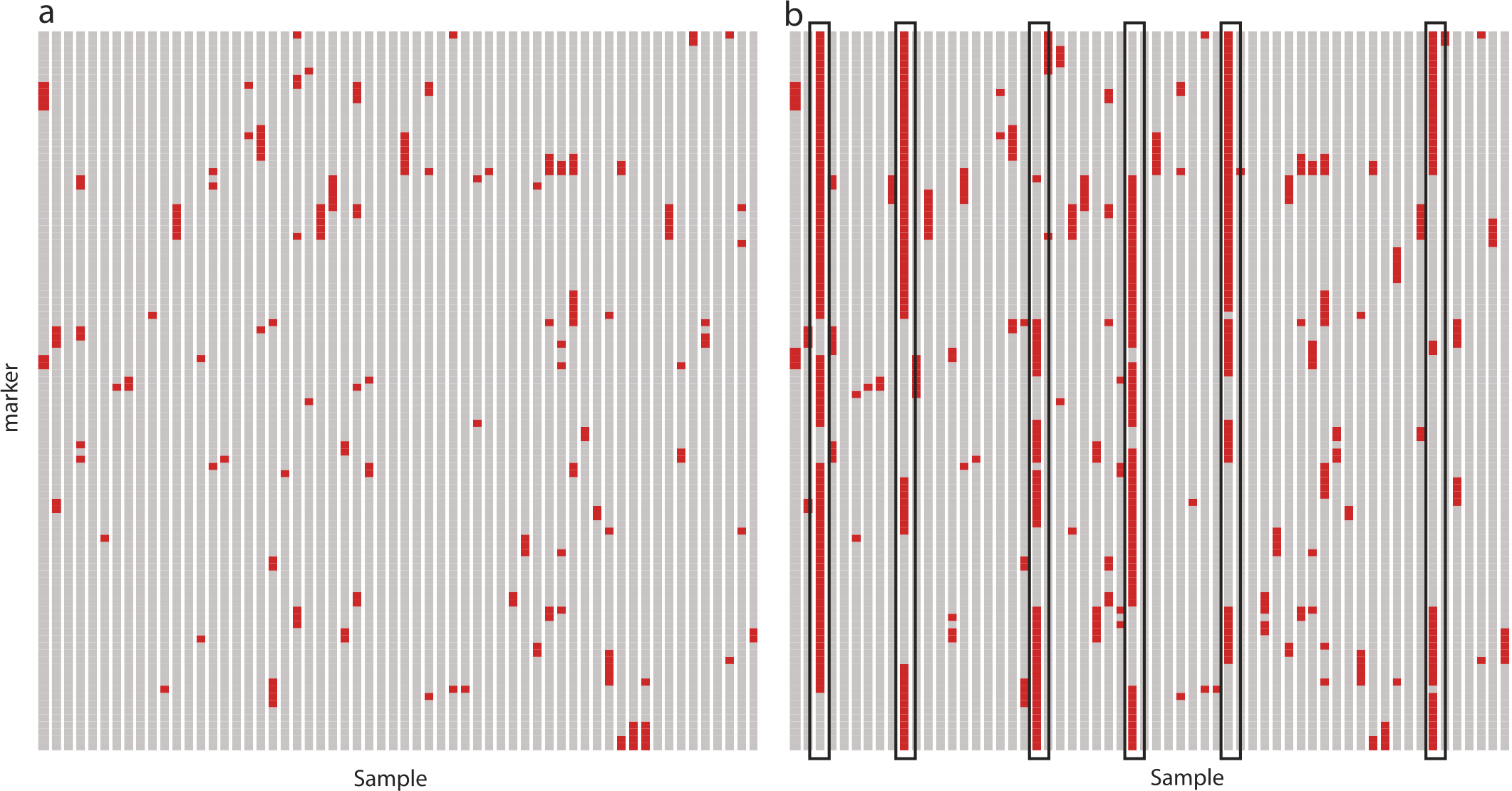

Supplement: S1 Fig — Experiment without a mislabeled sample (a) and with mislabeled samples (b). Grey and red correspond to values of 0 and 1 for the S element value, respectively. The rows represent markers along the genome and the columns represent different samples. (a) S values of 1 (red spots) are scattered in the figure, indicating that no sample has been mislabeled. (b) Vertical red bars indicate that the corresponding samples (columns) are very likely to have been mislabeled. In this simulated experiment, six (~10%) mislabeled samples sharing about 50% common marker genotypes with the correct sample were included, and these were all clearly identified. (PNG) [file pone.0171324.s001.png]

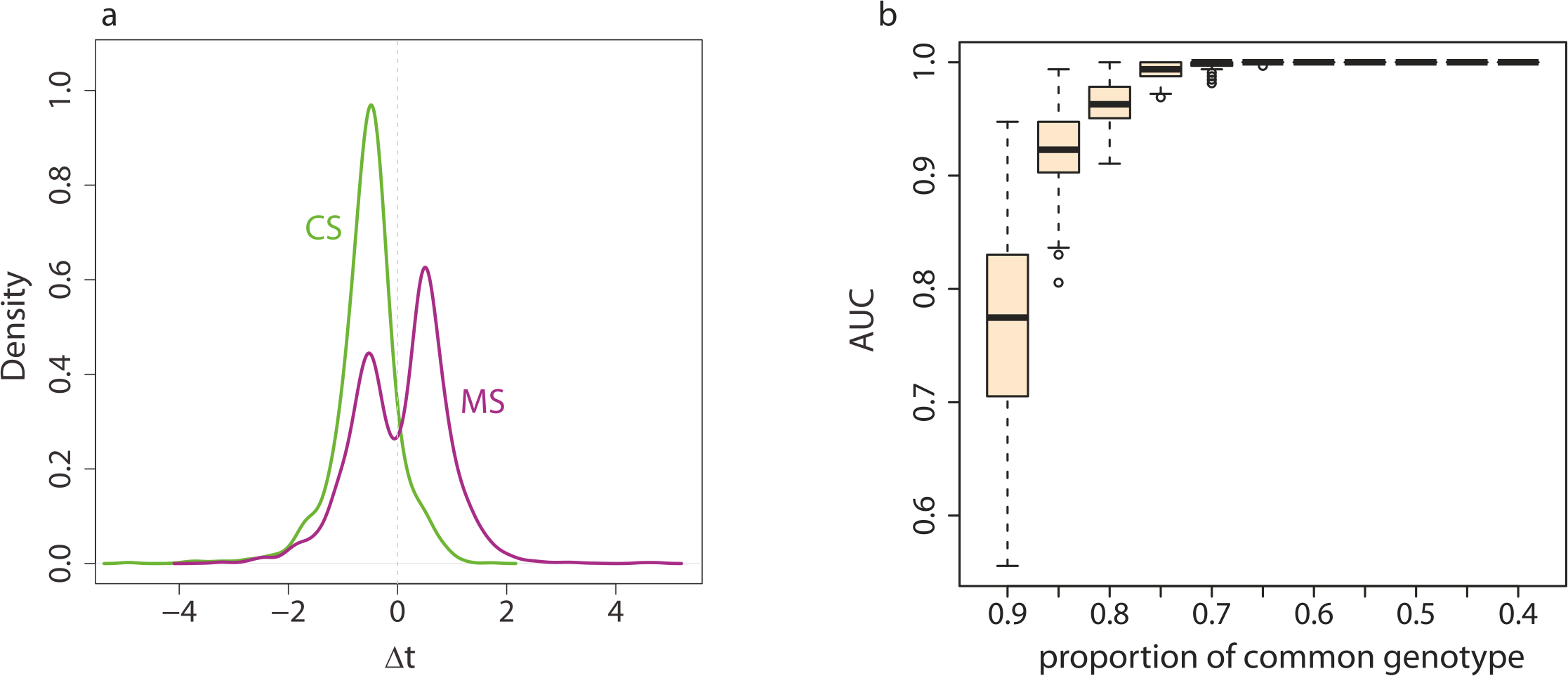

Supplement: S2 Fig — a) Comparison of the distribution of Δt for a correctly labeled sample (green) and a wrongly labeled sample (magenta) from a simulated dataset. The wrongly labeled sample and the true genotype are 70% identical. b) Box plot of the area under the receiver operating characteristic curve (AUC) for the reGenotyper algorithm for different scenarios. The x-axis represents ten different situations, i.e. different proportions of common marker genotypes between the mislabeled sample and the true genotype of this sample; in the most challenging scenario, the mislabeled sample shares 95% of its marker genotypes with the true sample. Under each scenario, 50 simulations were carried out. In each simulation, a gene expression dataset of 100 samples with 10% mislabeled samples was simulated. (PNG) [file pone.0171324.s002.png]

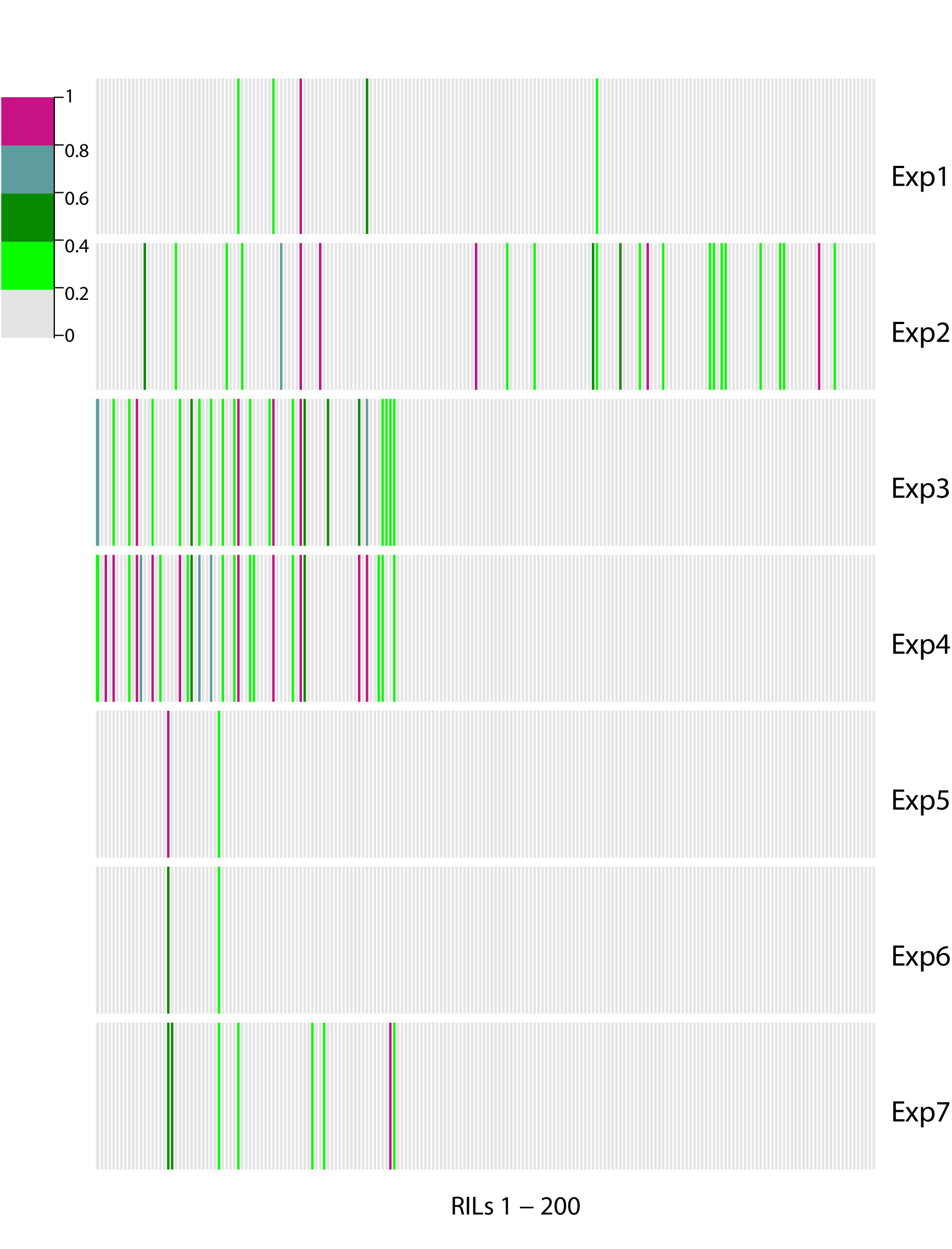

Supplement: S3 Fig — Sample across seven different experiments (rows) from our lab using the reGenotyper algoritm. Different shades of green gradients represent the mislabeling score. Darker green corresponds to higher scores, while magenta indicates that the sample was detected as MS with a score larger than 90%. White indicates that the sample was not selected for use in this experiment. (PNG) [file pone.0171324.s003.png]

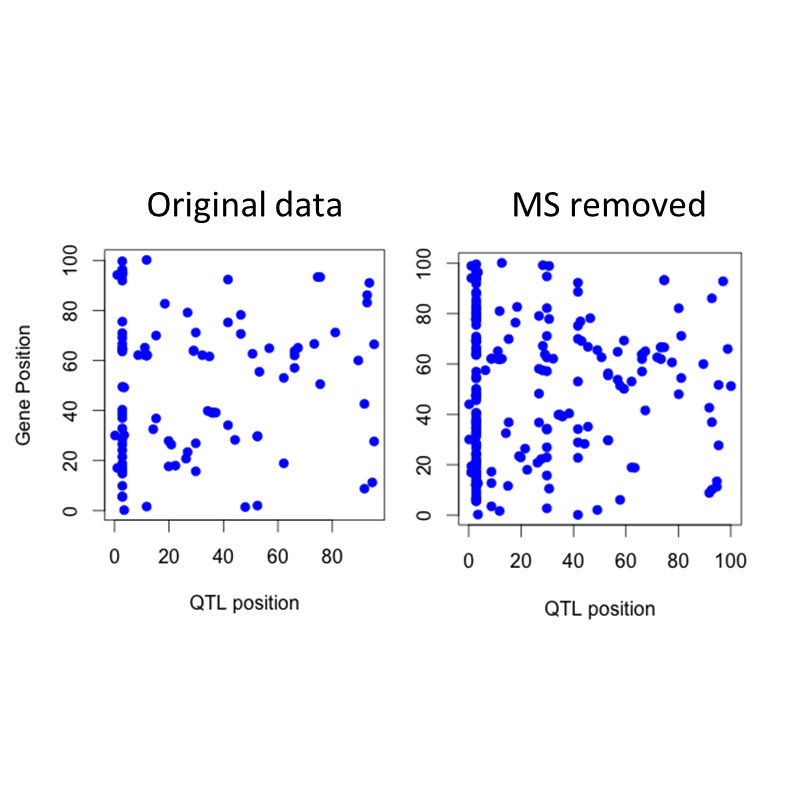

Supplement: S4 Fig — Figure compares results of QTL mapping on original data (left) and after removal of 5 MS (right). Removal of MS detected by reGenotyper results in sharp increase in number of QTLs shared between two temperatures used in the study (from 93 to 190–111%). (PNG) [file pone.0171324.s004.png]

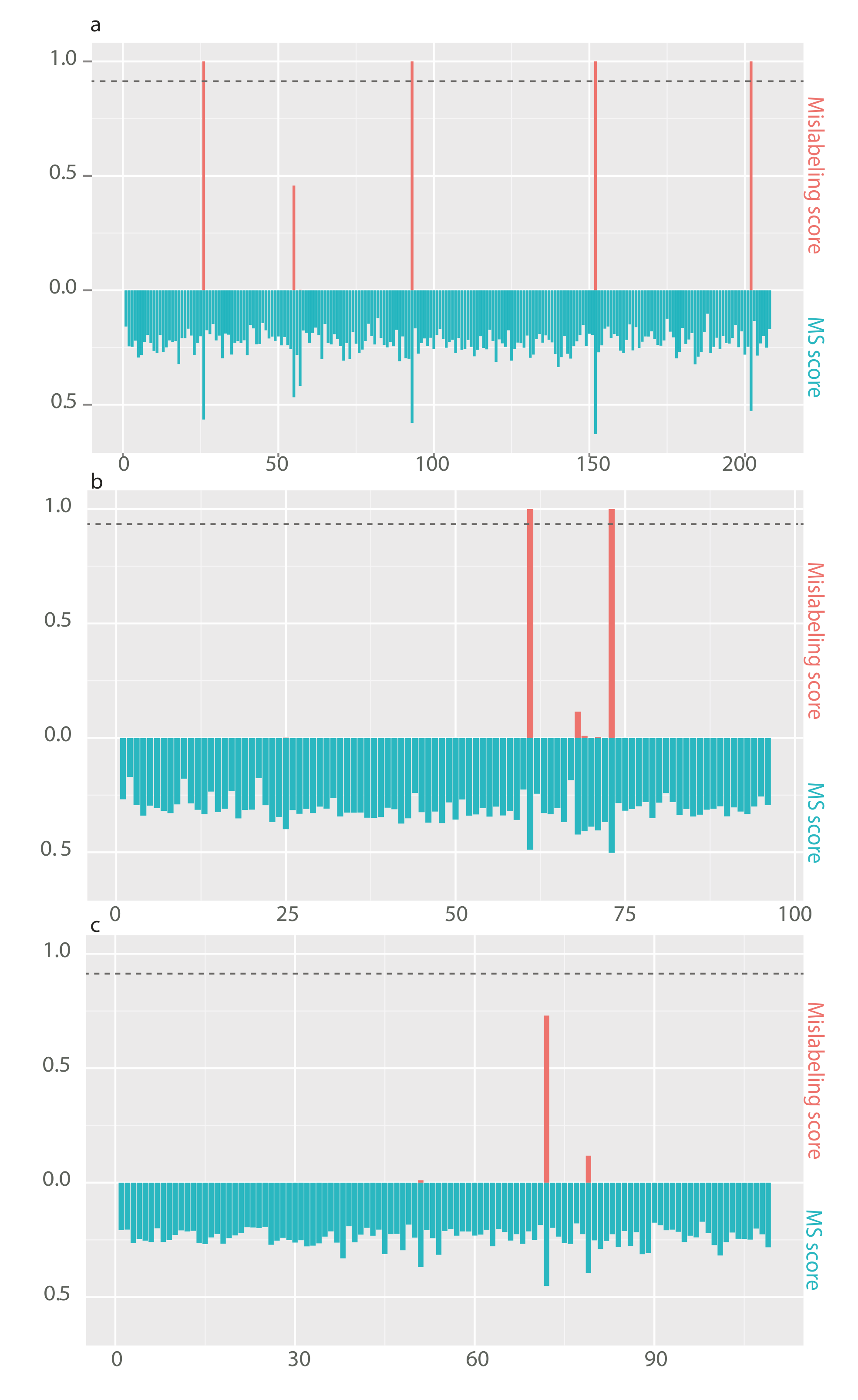

Supplement: S5 Fig — Respectively, 4 out of 208, 2 out of 96 and 0 out of 109 samples show a mislabeling score >0.9 (dashed line). (PNG) [file pone.0171324.s005.png]

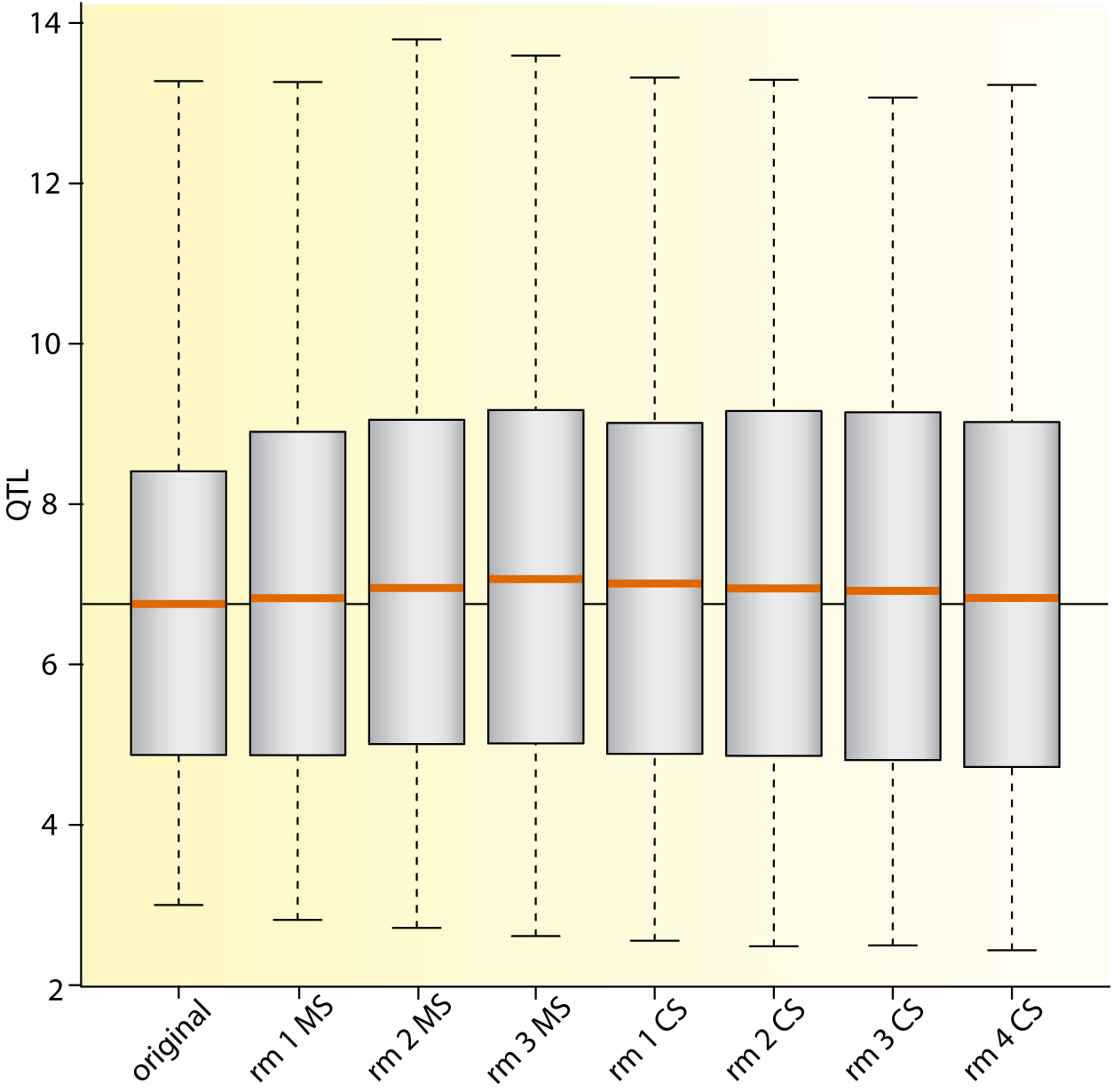

Supplement: S6 Fig — The genotype and phenotype data were simulated for 60 RILs in the same way as described in the S1 File. Three (5%) mislabeled genotypes were added to the data set. The samples were removed in decreasing order of their mislabeling score. First, three mislabeled samples (MS) and later samples with little chance of being mislabeled (i.e. low mislabeling score), correct samples, (CS) were removed. The median of detected QTLs (-log10P) significance distribution (orange line) increases after removing MS and drops again after removing CS. (x axis, rm = removing). (PNG) [file pone.0171324.s006.png]

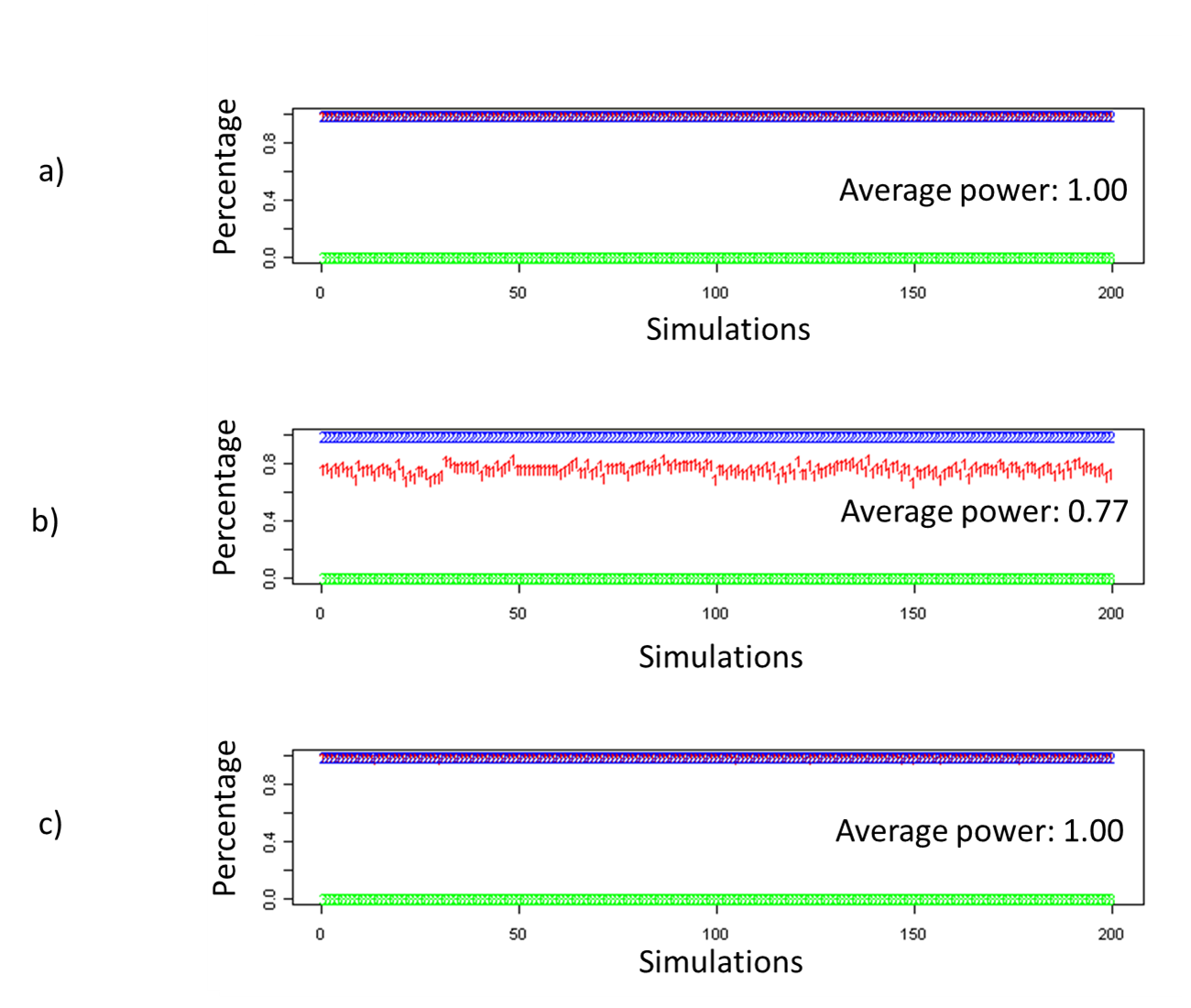

Supplement: S7 Fig — 200 simulations were performed and QTL mapping was performed a) before swapping samples to simulate mislabeling, b) after swapping samples and c) after swapping samples and recovering true genotypes using reGenotyper. Plots shows rate of: red 1 –phenotypes with simulated QTL that was mapped; blue 2 –phenotypes without simulated QTL, where no QTL was mapped; green 3 –phenotypes without simulated QTL, where QTL was mapped. After recovery with reGenotyper almost all of the QTLs could be mapped correctly, compared to 77% in case with MS. (PNG) [file pone.0171324.s007.png]
