# Supplementary material for: reGenotyper: Detecting mislabeled samples in genetic data
Source: PLoS One. 2017 Feb 13;12(2):e0171324. doi: 10.1371/journal.pone.0171324 (PMC5305221; doi:10.1371/journal.pone.0171324)
Supplement: S1 File — (DOC) [file pone.0171324.s008.doc]

# Supplementary results

## Algorithm for identifying mislabeled sample

The basic concept of the *reGenotyper* algorithm (S1 Fig) is a combination of a data perturbation strategy and majority rule:

Hypothesis I: The majority of samples are correctly labeled. This is generally valid in real experiments and guarantees the reliability of detected QTLs from the original data.

Hypothesis II: Perturbing the genotype at a particular marker of a mislabeled sample (wrong → correct) will often lead to an improved QTL significance for all molecular traits mapping to a QTL near that marker. This would be true for as many molecular trait–marker combinations as there are QTL, when the genotype of this sample has been wrongly labeled.

For recombinant inbred lines, perturbing means reversing the genotype from *A* to *B* or vice versa. For other crosses, such as F_2_ hybrids, perturbing can be implemented by changing the original genotype to one of the other possible genotypes at this marker, potentially taking the relative abundance of each genotype into account.

The *reGenotyper* algorithm (S1 Fig) works as follows:

For a sample *n*,

1. Compute the original QTL profiles (**Error! Objects cannot be created from editing field codes.**) for each molecular phenotype (*g* = 1,…*G*) at this marker *m* using the original genotype. Here, for illustration, a standard *t* statistics is used to assess QTL significance.
2. Select *K* molecular phenotypes with the most significant (top) QTL at this marker based on **Error! Objects cannot be created from editing field codes.**. For the illustrative analysis, *K* is chosen as 30 or 50, but in the *reGenotyper* software the user can specify a threshold that seems appropriate for their dataset. Generally, the *K* should not be too low as this could negatively affect the accuracy of our algorithm.
3. Perturb the genotype of this sample at this marker; compute the new *t* statistics for the selected top *K* molecular phenotypes
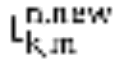
**Error! Objects cannot be created from editing field codes.**
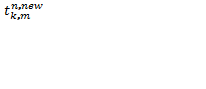
; then obtain the perturbed significance change value, which is defined as follows:

**Error! Objects cannot be created from editing field codes.**
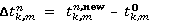
(*k* = 1,…, *K*) (1)

1. Summarize the perturbed significance change values. The summarized ∆*t* index for each sample *n*, denoted by S^n^, can be obtained by summing up the
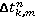
 **Error! Objects cannot be created from editing field codes.**from different sets of *K* molecular phenotypes at each marker *m*. To be more specific, for a given sample *n*, S^n^ is a vector of length *m*, which is defined as:

**Error! Objects cannot be created from editing field codes.** (2)

Where δ is a user-defined threshold.

Repeat steps 1-4 for all the markers (*m = 1, …, M*) available in the genotype data.

## Detecting the mislabeled sample(s)

S^n^ evaluates the correctness of the genotype for sample *n*, where the element
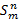
**Error! Objects cannot be created from editing field codes.** (*m* = 1,…, *M*) being 1 or 0 indicates the wrong or correct genotype, respectively. Since **Error! Objects cannot be created from editing field codes.**
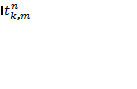
 for a certain phenotype *k* at a certain marker *m* might show a non-zero value simply by chance, the average of **Error! Objects cannot be created from editing field codes.**over the top *K* molecular traits at this marker *m* is used to determine**Error! Objects cannot be created from editing field codes.**. When the average of all **Error! Objects cannot be created from editing field codes.** values from *K* genes at one marker *m* exceeds a certain threshold δ, it suggests that the genotype at the marker *m* might be wrong and the *m*th element in the summarized ∆*t* index (**Error! Objects cannot be created from editing field codes.**) is set to 1. The threshold could be set at δ = 0, as obviously the expected average of all **Error! Objects cannot be created from editing field codes.** values for a correctly genotyped marker would be negative, or to a small positive value for a more conservative assessment (see below for a more detailed discussion of the expected distribution of **Error! Objects cannot be created from editing field codes.** values). In the examples, the threshold δ is set to 0, but users can define a more stringent threshold (e.g. δ = 0.1) depending on their prior belief about the frequency of wrongly labeled samples and the cost of missing a wrong label.

For each sample *n* (*n* = 1, …, *N*), S^n^ can be computed based on equation (2). The sample *n* is considered to be a potentially wrongly labeled sample if the number of markers with a summarized ∆*t* index S of 1 exceeds a certain empirical threshold (such as 30% of all markers), particularly when those elements show up in blocks rather than being scattered along the genome (S1 Fig). The reason for this property lies in the fact that the recombination frequency in RILs is relatively low, and neighboring markers tend to be linked across larger distances.

## Recovering the true genotype for the mislabeled sample based on S^n^

Based on the information from the summarized ∆*t* index (**Error! Objects cannot be created from editing field codes.**), it is straightforward to estimate the true genotype for those samples detected as mislabeled: the original genotype is reversed (*A* becomes *B*, and vice versa) at all markers with an**Error! Objects cannot be created from editing field codes.** value of 1. When it is known that the detected mislabeled samples were caused by sample swapping within a RIL collection, it is possible to find the most likely correct label by replacing the genotype of mislabeled samples by that of each of the other RILs in turn. The RIL with the largest sum of S^n^ (i.e. a sample with the highest number of markers with an **Error! Objects cannot be created from editing field codes.** value of 1) is the best candidate for the incorrect label. Based on this, reGenotyper can also be employed in an iterative manner; i.e. after detecting the first mislabeled sample, the most likely correct genotype can be immediately recovered, either by comparison to the existing RIL collection or by choosing the true genotype directly. Subsequently, the recovered genotype is further used to detect the second sample most likely to have been mislabeled. In this way, the power of reGenotyper for detecting the mislabeled samples is increased further. Additionally, our algorithm can be employed in a pair-wise manner, i.e. all possible pairs of samples are evaluated to estimate the chance of them containing mislabeled samples, in order to obtain the combined evidence for pairs of samples being mislabeled. Such a combined analysis is particular powerful for detecting swapped samples that have relatively similar genotypes.

## Calculating mislabeling score using permutations results

The reGenotyper algorithm, using the summarized ∆*t* index S^n^, relies on the concept that perturbing the genotype of a mislabeled sample leads to an increased QTL significance for many molecular traits mapping to a QTL close to that marker, and this holds for many gene–marker combinations, when there are QTL near the markers. Clearly, ∆*t* values evaluate the significance difference with respect to a data perturbation (one perturbing at this specific marker). The elements in S^n^ are the summarized ∆*t* values per gene–marker combination, and a binary value (1 or 0) is assigned for sample *n* depending on a pre-defined threshold. This can be further improved by using all ∆*t* values from all trait–marker combinations, i.e. the distribution of these ∆*t* values.

When the sample has been correctly labeled, the distribution of all ∆*t* values, from all the trait–marker combinations after perturbing the genotype of this sample, would roughly show a single component distribution and the median of the distribution is expected to be smaller than zero. In contrast, if a sample was originally mislabeled, the distribution of ∆*t* values for this sample *n* ($\Delta t_{k,m}^{n}$ values from *K* traits at all markers) would show a mixture distribution, with one extra component with a mean larger than zero (S2 Fig a). This is because perturbing the genotype of this mislabeled sample leads to the true genotype, which results in the real maximal significance for the QTL. Although ∆*t* for a certain trait at a certain marker might show a non-zero value simply by chance, the identification of a mislabeled sample is based on the ∆*t* values from a large number of traits measured in parallel (e.g. the top 50 QTLs at one specific marker) at a number of marker positions (different sets of QTLs at those markers), thus making use of the special data-richness of genetical genomics experiments. In other words, the distribution of all $\Delta t_{k,m}^{n}$ ( *k* = 1,…, *K*, *m* = 1,…, *M*) from one sample is actually used to evaluate the chance that it was mislabeled. This is summarized by calculating the area of ∆*t* larger than zero, referred to as the “mislabeled sample score” (MS) in the density plot (S2 Fig a):

$MSscore=\sum_{m}^{M} \sum_{k}^{K} \Delta t_{k,m}^{n}$ (3)

Clearly, the larger the MS score, the more likely it is that this sample was mislabeled.

In order to evaluate the chance of a sample having been mislabeled, and to avoid false positive results caused by potential outliers in the data, a permutation strategy is employed to obtain the distribution of MS scores for a wrongly labeled sample. When using permutations, we randomly select a small subset of samples (e.g. 5% [this can be specified by the user, based on their expectations about the reliability of their labels]) out of all those used in the experiment, where each sample has an equal chance of being selected. Then the genotypes of the selected samples are randomly permuted, which eventually will create wrongly labeled samples in the genotype data while keeping the phenotype data unchanged. By doing this 1000 times, we create a large number of datasets, for which we can compute the MS scores of those intentionally wrongly labeled samples for each of the datasets. Thus we get an estimate of the reference (null) distribution of MS scores.

Raw MS score is dependent on the data structure and does not provide statistical significance for mix-up. However, with reference distribution we know what MS scores we could expect given our data structure and a certain percentage of sample mix-ups. We calculate the percentage of permuted scores that is lower than an MS score for the current sample; this is called the mislabeling score. For example, to achieve a mislabeling score of 0.9 a sample needs to have an MS score higher than 90% of permuted samples out of 1000 permutations.

## Collective evidence for a mislabeled sample in the replicated experiments

When the same individuals or populations are used in multiple experiments, as is often the case for genetical genomics studies based on recombinant inbred lines, the rank product technique [1] can be employed to jointly evaluate the chance of a sample being wrongly labeled (assuming that the same labeling mistakes are shared between experiments). When only some of the samples are repeatedly used across experiments, the average ranking is more appropriate to evaluate the joint evidence of a mislabeled sample.

## Comparing mislabeled samples and correct samples

### a) Simulation of a genetical genomics experiment without mislabeled samples

We first simulated marker genotypes for a genome of 5 chromosomes, each with a length of 100 centi-Morgan and containing 100 markers at evenly spaced intervals. Then, 500 gene expression profiles were simulated with one QTL per transcript for 60 RILs. Measurement errors were randomly and independently drawn from normal distributions with a mean of 0 and standard deviation of 0.7. The integrated ∆*t*R Rindex for each sample, denoted by S, was computed according to equation (2). From the result (S1 Fig a), most RILs can have S values of 1 (red) at a few marker positions by chance, but the large majority (98%) of S values are 0 (green), indicating that there is no mislabeled sample in the data.

### b) Simulation of a genetical genomics experiment with mislabeled samples

The genotype and phenotype data were simulated for 60 RILs in the same way as described in a). The genotypes of six samples (10%) were manually swapped (i.e. exchanged between two samples that were both included in the dataset). As can be seen in S1 Fig b, the majority of RILs have mostly S values of 0 (green) while six RILs show S values of 1 (red) at a large number of marker positions in large blocks along the genome (visible as vertical red bars). These cases exclusively correspond to the simulated mislabeled samples.

# References

1. Rockman, M.V., Skrovanek, S.S. & Kruglyak, L. Selection at linked sites shapes heritable phenotypic variation in C. elegans. *Science* **330**, 372-6 (2010).
2. Gerrits, A. *et al.* Expression quantitative trait loci are highly sensitive to cellular differentiation state. *PLoS Genet* **5**, e1000692 (2009).
3. Brem, R.B. & Kruglyak, L. The landscape of genetic complexity across 5,700 gene expression traits in yeast. *Proc Natl Acad Sci U S A* **102**, 1572-7 (2005).
